# Design and Evaluation of a Contextual Model for Information Retrieval From Web-Scale Discovery Services to Improve Evidence-Based Practice by Health Care Practitioners: Mixed Methods Study

**DOI:** 10.2196/12621

**Published:** 2019-08-21

**Authors:** Alvet Miranda, Shah Jahan Miah

**Affiliations:** 1 Victoria University Business School Victoria University Footscray Park Campus Footscray Australia

**Keywords:** information retrieval, design science research, Web-scale discovery, evidence-based practice, libraries, digital, artefacts

## Abstract

**Background:**

Practicing evidence-based health care is challenging because of overwhelming results presented to practitioners by Google-like Web-scale discovery (WSD) services that index millions of resources while retrieving information based on relevancy algorithms with limited consideration for user information need.

**Objective:**

On the basis of the user-oriented theory of information need and following design science principles, this study aimed to develop and evaluate an innovative contextual model for information retrieval from WSD services to improve evidence-based practice (EBP) by health care practitioners.

**Methods:**

We identified problems from literature to support real-world requirements for this study. We used design science research methodology to guide artefact design. We iteratively improved prototype of the context model using artificial formative evaluation. We performed naturalistic summative evaluation using convergent interviewing of health care practitioners and content analysis from a confirmatory focus group consisting of health researchers to evaluate the model’s validity and utility.

**Results:**

The study iteratively designed and applied the context model to a WSD service to meet 5 identified requirements. All 5 health care practitioners interviewed found the artefact satisfied the 5 requirements to successfully evaluate the model as having validity and utility. Content analysis results from the confirmatory focus group mapped top 5 descriptors per requirement to support a true hypothesis that there is significant discussion among participants to justify concluding that the artefact had validity and utility.

**Conclusions:**

The context model for WSD satisfied all requirements and was evaluated successfully for information retrieval to improve EBP. Outcomes from this study justify further research into the model.

## Introduction

### Background

**Figure 1:**
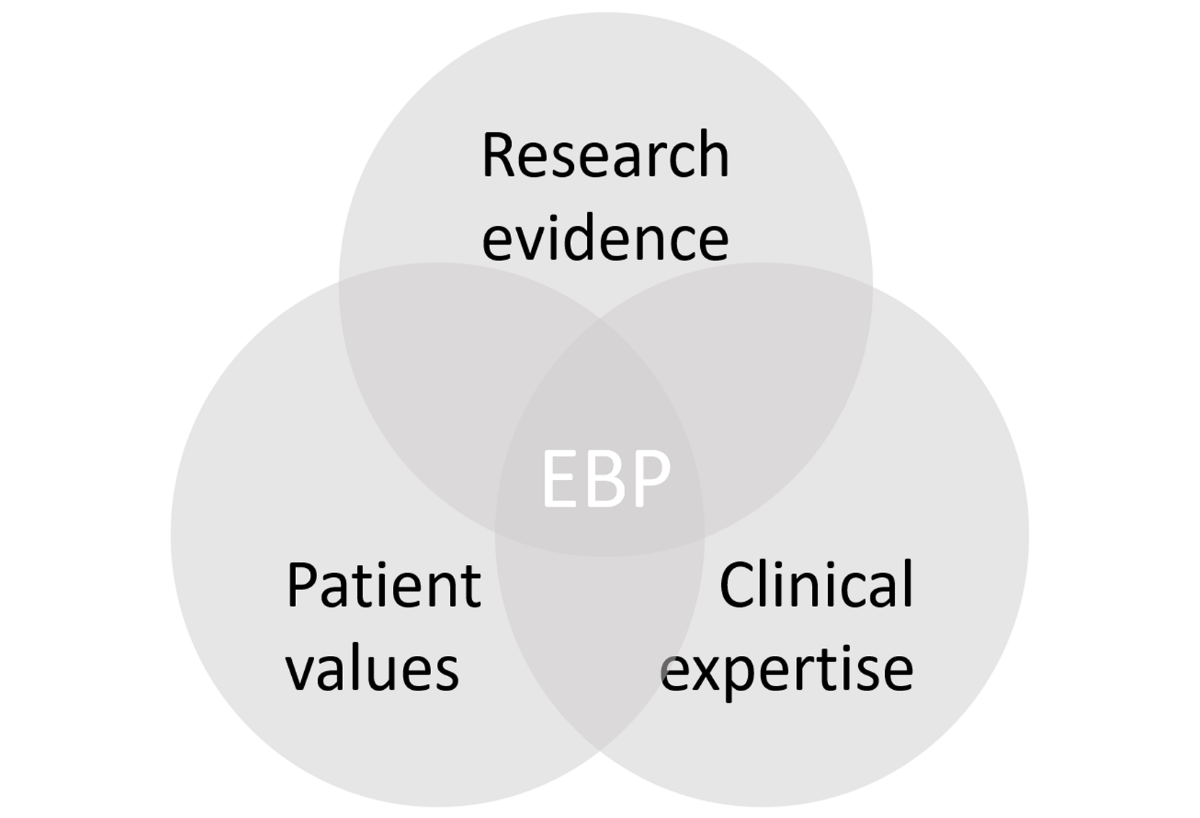
Three components of evidence-based practice.

Evidence-based practice (EBP) represents an amalgamation of research evidence, clinical experience and expertise, and patient values or preferences in the process of clinical patient care [1], as shown in Figure 1. This study introduces a contextual approach to research evidence modeled using design science research (DSR) [2] so health care practitioners can improve EBP.

A positive correlation was found between clinician’s behavior and utilization of research evidence from information systems as identified by a study involving 439 nurses and physicians from public and private hospitals [3]. The study, however, concluded that more support is needed for health care practitioners to use research evidence systems.

**Figure 2:**
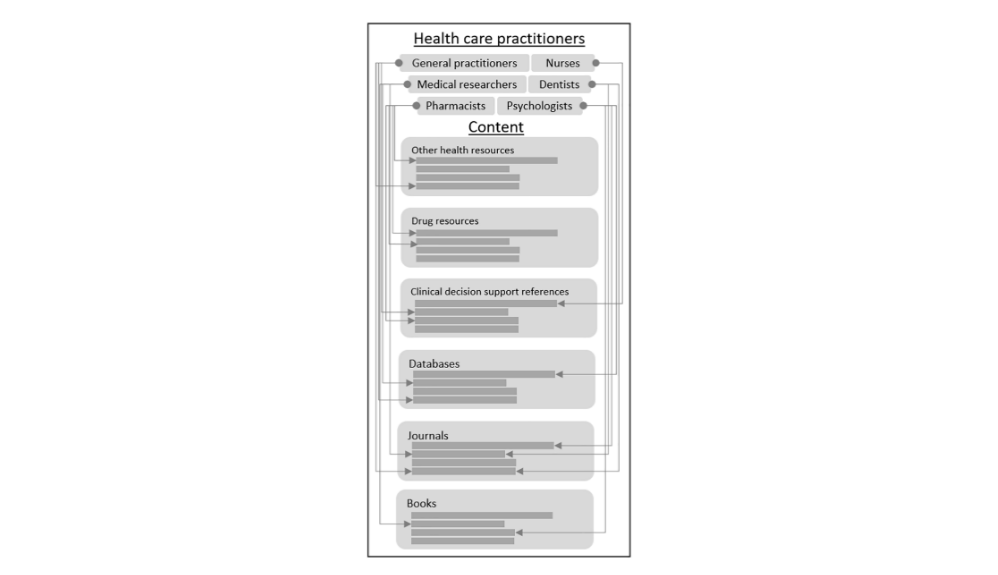
Health information.

There is a myriad of evidence-based clinical information resources across various publisher platforms and use of these resources varies from practitioner to practitioner as illustrated in Figure 2.

Electronic resources (e-resources) themselves are classified as journals, books, databases, and clinical decisions support references, and each classification has many associated publishers, vendors, and electronic service providers. Being aware of and retrieving information from all these e-resources was challenging for clinicians who needed information for patient care, medical research, and professional growth [4].

At the turn of the 21st century, this problem was solved to an extent by federated search systems; however, they were unreliable and slow as they connected with each publisher platform in real time, causing users to wait several minutes to see results or miss results entirely when connectors failed [5].

Evidence-based clinical information is traditionally licensed content inaccessible to Web search engines, so the information retrieval gap was satisfied by Web-scale discovery (WSD) services starting with OCLC’s (Online Computer Library Center) WorldCat Local in 2007, Summon from Serial Solutions in mid-2009, EBSCO Discovery Service (EDS) in 2010, followed by Ex Libris Primo Central and Innovative interfaces Encore Synergy [6].

**Figure 3:**
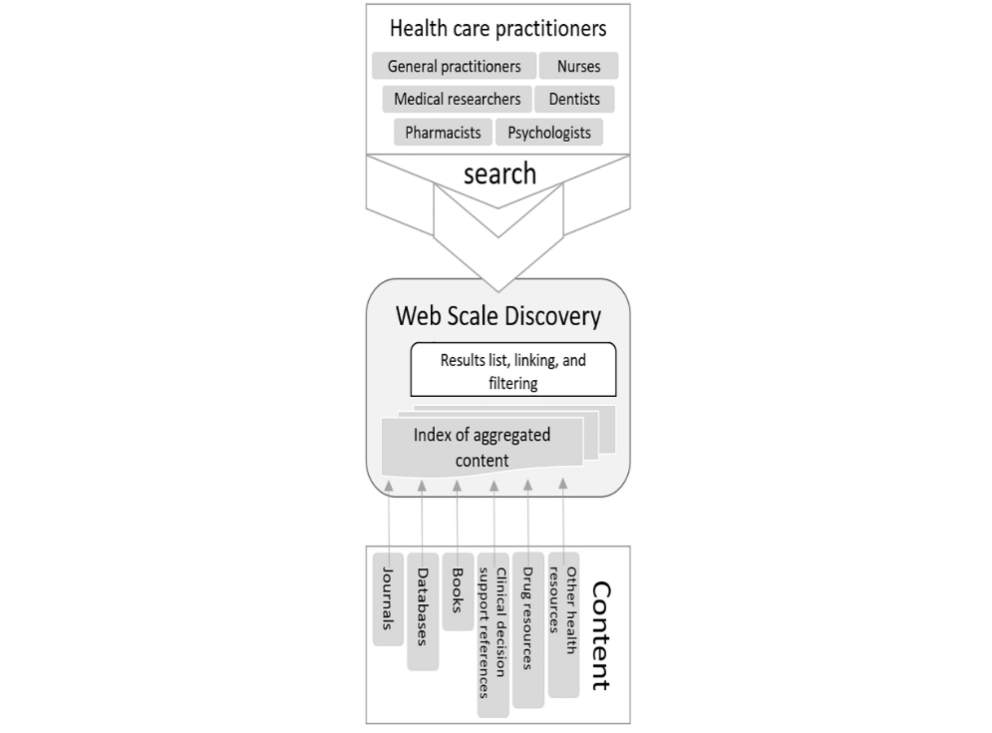
Health care practitioner’s retrieving information from a Web-scale discovery.

WSD service–based solutions offer user-friendly search interfaces, relevance ranking, and large, centralized indexes, allowing rapid, simultaneous searching [7]. Figure 3 draws upon Figure 2 and illustrates how a WSD solved the issues of fragmented and unknown e-resources by indexing licensed content metadata and making it retrievable by clinicians using a single interface on the screen. By pre-harvesting content from myriad databases into a single index, WSD tools improve on federated searching tools’ speed, de-duplication abilities, relevancy rankings, and the amount of content that can be accessed [8]. WSD services are used worldwide; for example, the EDS is used in over 100 countries across 11,000 institutions [9].

### Problem Identification and Motivation for Research

Focusing on the medical sector, a health science research comparing WSD from 3 vendors concluded that none of the services were overwhelmingly more effective in retrieving relevant information, placing them at 50% to 60% likelihood an average user would find adequate resources [7].

When introducing WSD, a study by Hoy uncovered various disadvantages that needed to be addressed further through new research [10], namely, (1) searches produced too many results overwhelming users, (2) object types and formats caused confusion, (3) users experienced noise, and (4) users needed help using limiters to retrieve specific information.

There are also challenges in offering the same set of e-resources to all clinician groups, as a study [11] comparing the information delivery needs of physicians and nurses found significant discrepancies with wide gaps in behavior and motivation as physicians accessed different online resources from nurses for clinical practice.

Articles from the *Journal of Medical Internet Research* since 2010 discussed research evidence and barriers faced by health care professional to find, appraise, and apply emerging evidence at the point of delivery [12]. As a means to improve EBP adoption rates, health care organizations adopt several strategies such as local consensus processes, distribution of education material, outreach visits, and reminders; however, lack of time, perceived difficulty, and nonintuitive platforms are cited as issues [13]. Web-based knowledge resources had significant impact on access to evidence-based information, which positively correlates with the growth in usage of WSD services over the 2010 decade [14].

It can be deduced by comparing Figure 2 and Figure 3 that although WSDs gave health care practitioners a single point of entry for information retrieval, the array of aggregated content becomes problematic for individuals and clinical groups requiring context they would otherwise get by using the various content platforms directly.

### Aims and Solution Objectives

This research aimed to develop and evaluate a model [15] to improve information retrieval from WSD services so health care practitioners can better adopt EBP by satisfying research evidence needs. The model introduces a context layer to existing WSD services to reduce the ambiguity of information by pre-capturing and integrating clinician’s context during the information retrieval process [16].

**Figure 4:**
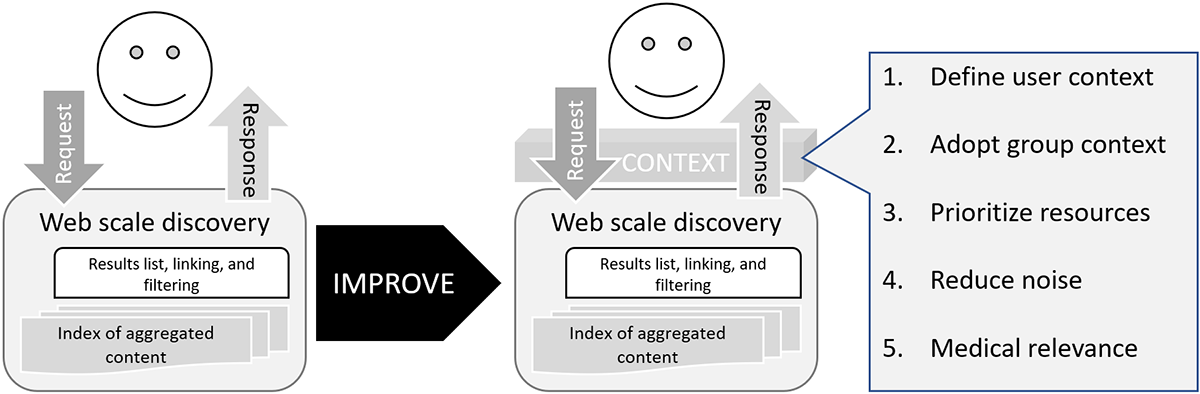
Improving Web-scale discovery using contextual artefact or model.

The contextual layer improves WSDs having a computer science perspective to a knowledge formulation or acquisition system based on the theory of information need for information retrieval by Cole connecting information to knowledge [17]. Figure 4 focuses on the WSD part in Figure 3 and illustrates the improvement by adding a context layer between the user and the WSD that meets requirements listed in Table 1 derived from the literature referenced above.

**Table 1:** Context model requirements.

No.	Requirement	Description
R1^a^	Define user context	Allows a clinician to select settings and configuration to define their context to apply when querying a WSD^b^
R2^c^	Adopt group context	Adopt a clinical group context to receive similar settings to other health care practitioners. Adjust to user level if necessary
R3^d^	Prioritize resources	Display the resources that are most important to the clinician higher up the result list based on applied context
R4^e^	Reduce noise	Applying context to queries with WSD services should reduce noise from resources not applicable to the clinician
R5^f^	Medical relevance	Improved relevance of information presented to the clinician after the context is applied

^a^R1: Define user context.

^b^WSD: Web-scale discovery.

^c^R2: Adopt group context.

^d^R3: Prioritize resources.

^e^R4: Reduce noise.

^f^R5: Medical relevance.

### Literature Review

This section reviews prior work including problems and findings from practice relevant to this research. It also explores prior knowledge and artefacts attempting to solve similar problems to scope knowledge gap. Finally, a literature-based conceptual framework is developed to guide this research.

#### Problems With Web-Scale Discovery Services for Evidence-Based Practice

Finding and retrieving research evidence is an essential part of EBP as shown in Figure 1, but a relevant study [4] found that the top 3 barriers to EBP besides time were unfamiliarity with bibliographic databases, difficulty accessing research material, and not seeing the value of research for improving practices.

To minimize these barriers, WSD services offer large centralized indexes of many resources, a relevance ranking algorithm to find information, and a user-friendly interface offering a Google-like experience. Although adoption of WSD services in academia and the public sector is high, their uptake in medical institutions has been slim [7].

Mayo clinic based in the United States is a health care institution that looked to adopt a WSD and published the results from their analysis as they evaluated the “promise to deliver quick, efficient and comprehensive search experience through a single-entry point.” The Mayo Clinic WSD workgroup concluded to forgo purchasing a WSD service at the time as their collection and users are heavily concentrated within the medicine and health care disciplines. Necessary requirements to meet their user demands and expectations lacked; however, the study acknowledged any future evaluation should consider a trial because of the customizable nature of the services [18].

In addition to health care–specific information needs, a study into research information needs and barriers [19] found that there were significant differences in needs between a primary care physicians and hospital-based physicians as they have different types of consultations. To add to the complexities of information needs, researchers compared accessing online databases between physicians and nurses to conclude that the behavior and motivations varied significantly between the 2 groups [11]. This is a problem for WSD services as they offer a single point of entry for all health care practitioners and rely on the relevance algorithm, which is not sufficient as established by Hoy’s research [10].

#### Prior Artefacts to Solve Problems

A study comparing 3 WSDs for health care sciences found that none of 3 WSD services—Ex Libris Primo, ProQuest’s Summon, and EDS—were overwhelmingly more effective in returning relevant results for health sciences research specifically [7].

According to a study looking at information needs of health care practitioners caring for cancer patients, a range of information systems were developed, but most failed to meet the information needs. A possible reason in their opinion was the systems were developed without respecting and analyzing the information needs of the practitioners [20].

Narayanan, in an article published in December 2017, reviewed the current state of WSD services including the services mentioned before plus OCLC WorldCat Discovery services and concluded that although they have had a positive impact, user-centered requirements such as relevancy and personalization were not present [21].

In the past, however, user-centered or context-sensitive information seeking was attempted by systems with a smaller dataset compared with WSD services. For example, Saparova piloted a federated search system that factored in information needs of 51 clinicians. Clinicians found the search system easy and intuitive to use; however, a key piece of feedback was personalization features as the study concluded that successful adoption of a clinical information system depends on its human, technology, and organization fit [22].

In the book *Implementing Web-Scale Discovery Services—A Practical Guide for Librarians*, Thompson details the levels of customization and configuration capabilities available in the WSD services using the back-end administration tool [23]. This, however, does not satisfy the user and group context requirements, but it eludes to the possibility that a context model could be implemented to extend or improve information retrieval from WSD.

#### Requirements Gap

The above literature demonstrates that significant research was conducted to determine the state of WSD services to facilitate EBP by health care practitioners and suggests that information retrieval systems lacked functions to meet the requirements of this research. Table 2 demonstrates the functional gap across systems identified in the literature.

**Table 2:** Requirements gap.

Previous studies to solve information retrieval	R1^a^	R2^b^	R3^c^	R4^d^	R5^e^
EBSCO Discovery Service	No	No	No	Yes	Yes
Proquest Summon	No	No	No	Yes	Yes
ExLibris Primo (ExLibris was acquired by Proquest)	No	No	No	Yes	Yes
OCLC^f^ WorldCat Discovery	No	No	No	Yes	Yes
Federated Search System [22]	No	No	Yes	Yes	Yes

^a^R1: Define user context.

^b^R2: Adopt group context.

^c^R3: Prioritize resources.

^d^R4: Reduce noise.

^e^R5: Medical relevance.

^f^OCLC: Online Computer Library Center.

### Conceptual Framework

**Figure 5:**
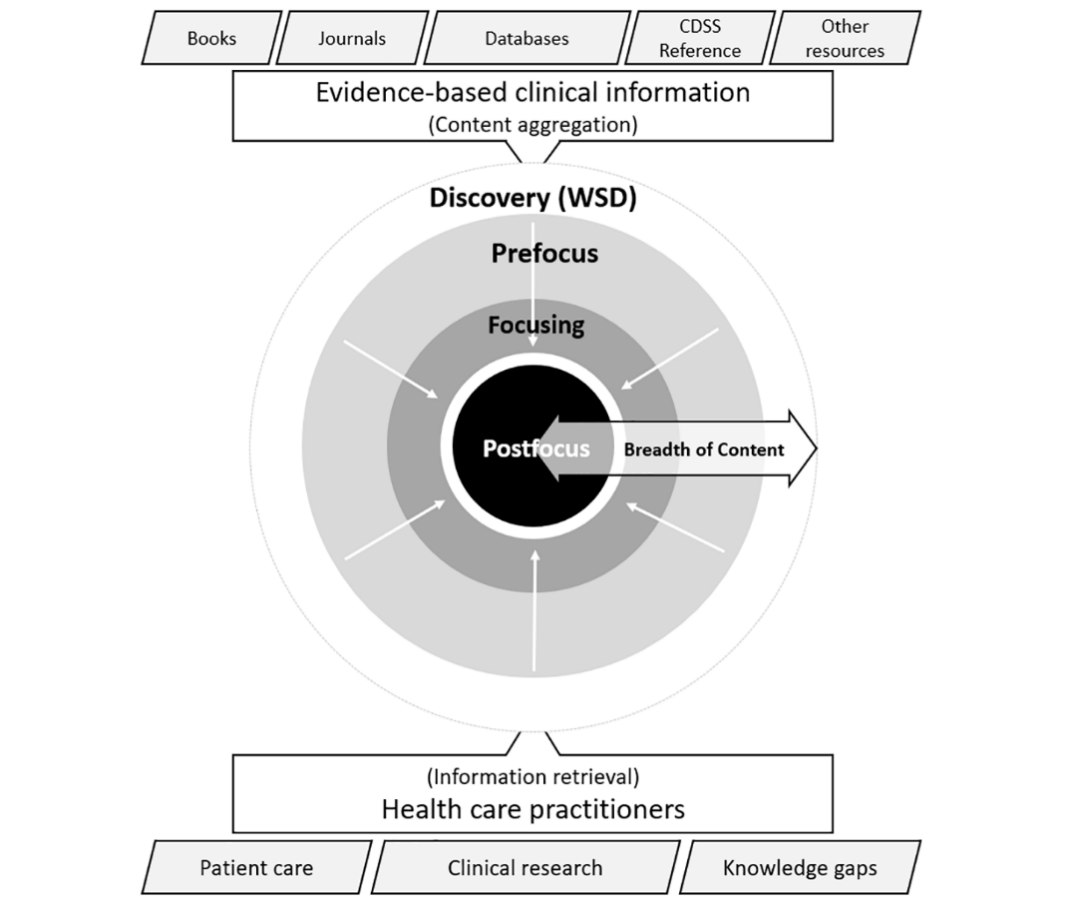
Theory of information need applied as context to Web-scale discovery. CDSS: WSD: Web-scale Discovery

Using a theory of information need, this research looks to approach developing the solution artefact differently compared with previous attempts discussed in the literature review. Health care practitioners use evidence-based clinical information to deliver patient care, conduct clinical research, and fill knowledge gaps. The WSDs are central to connecting clinicians’ information needs to knowledge, and the literature reveals the 2 primary functions of WSDs, which are aggregation of resources or knowledge and facilitate searching using algorithms. A contextual layer is needed to improve information retrieval, and for this we look to Cole’s theory of information use. Figure 5 shows how the following types are layered inward such as peels of an onion:

1. Prefocus: User borrows an existing frame for a new topic, and this frame can be based on adjacent topics, analogies, or knowledge stored in memory.
2. Focusing: Focus formulates, and the topic manifests its own frame.
3. The information need frame is fully developed to govern searching the topic.

The fulfillment of the prefocus, focusing, and postfocus inward journey of the user will help form the contextual framework necessary to improve information retrieval from WSDs. Applying these attributes to the context model is central to incorporating a user-oriented lens to this research as highlighted by Cole [17].

**Figure 6:**
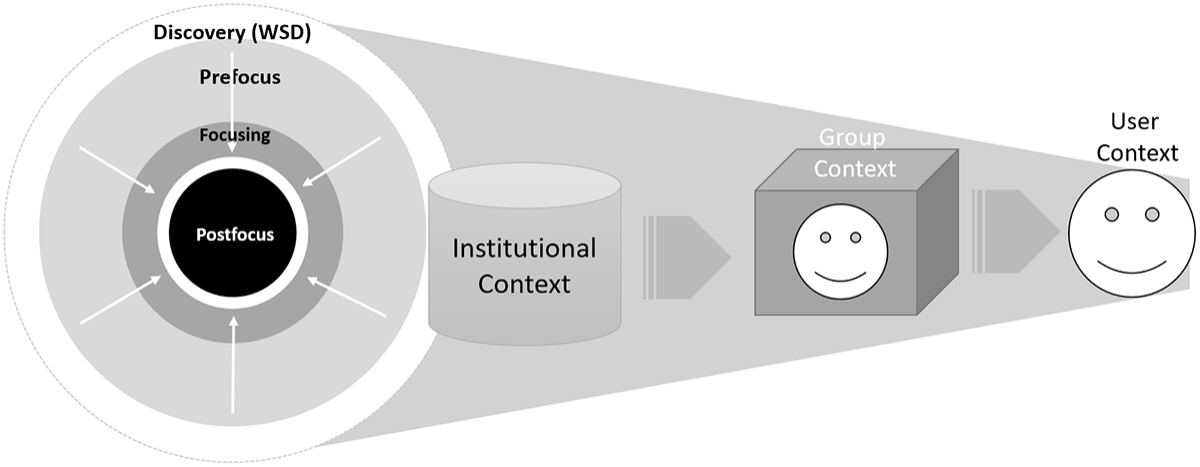
Nature of Web-scale discovery context at institution, group, and user level.

While reviewing implementation of WSD services, it is evident that these systems can be customized; however, the context is limited to the institutional level. A key aim of this research is to improve the current systems and offer personalization or context at the clinical group and user level as illustrated in Figure 6.

## Methods

### Paradigm

The study adopted a constructivism paradigm to maintain an appropriate research philosophy [24]. It is a paradigm that encourages intuitive thinking and guesswork from researchers who should discover and learn principles, facts, and concepts for themselves.

### Design Science Research

This research will use DSR methodology (DSRM), which falls under the constructivist paradigm to develop a solution artefact model as part of a larger context [25] to solve the problems described earlier in this study. In the case of this research, the artefact or context model created to address the problem [26] is an innovative approach that applies context to information retrieval from WSDs to improve research evidence as part of EBP.

**Figure 7:**
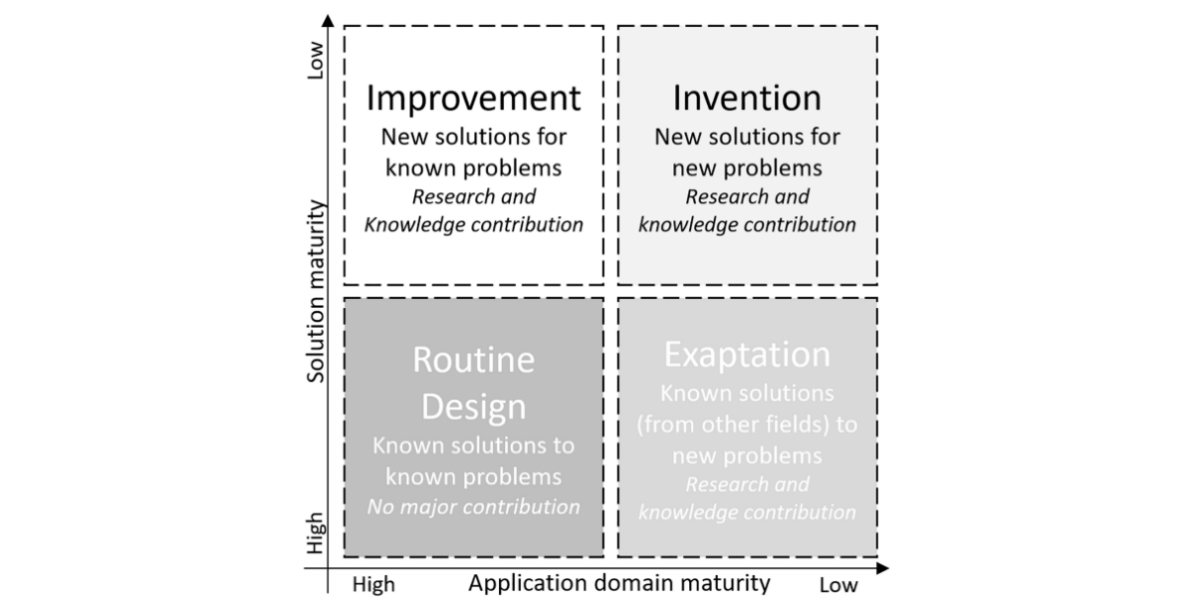
Knowledge contribution framework.

Referring to the knowledge contribution framework in Figure 7, this research falls under the improvement quadrant [27] as it addresses a known problem clinicians and health care professional face while retrieving evidence-based information from WSDs for EBP by developing an innovative artefact model prescribing a contextual approach to retrieving results from WSDs.

The use of DSRM for improvement is a proven principle based on several case studies [2].

**Figure 8:**
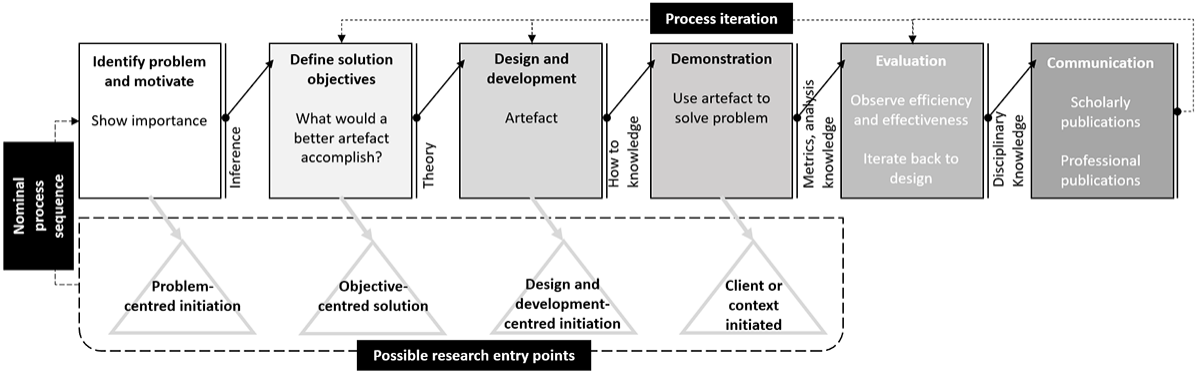
Design Science Research Methodology derived from Peffers (2014).

The DSRM illustrated in Figure 8 consists of 6-step nominal process sequences that interact iteratively based on findings from subsequent processes [2]. During step 1, the problem is identified along with motivation for a solution, described in the Introduction section of this study.

This is followed by defining solution objectives (Aims section of this study), which serves as inputs to the design and development for creating the artefact. Versions of the artefact are developed through iterative prototyping [28] and used to solve the problem in the demonstration step.

The evaluation step involves determining the artefact’s usefulness based on validity, utility, quality, and efficacy. This research uses the technical risk and efficacy strategy [29] for iterative artificial formative evaluations. Naturalistic summative evaluation via convergent interviewing [30] was used to determine the validity and utility of the artefact with 5 health care practitioners who each were practicing actively for over 15 years. A confirmatory focus group [31] consisting of 5 doctoral level health researchers doing academic research within digital health care for at least 3 years was also conducted, and the transcribed content was analyzed quantitively [32] to ensure significant discussion to support the group’s conclusion.

Findings from this study are communicated using recommendations by Gregor [27] to contribute to the existing body of knowledge.

### Artefact Description

**Figure 9:**
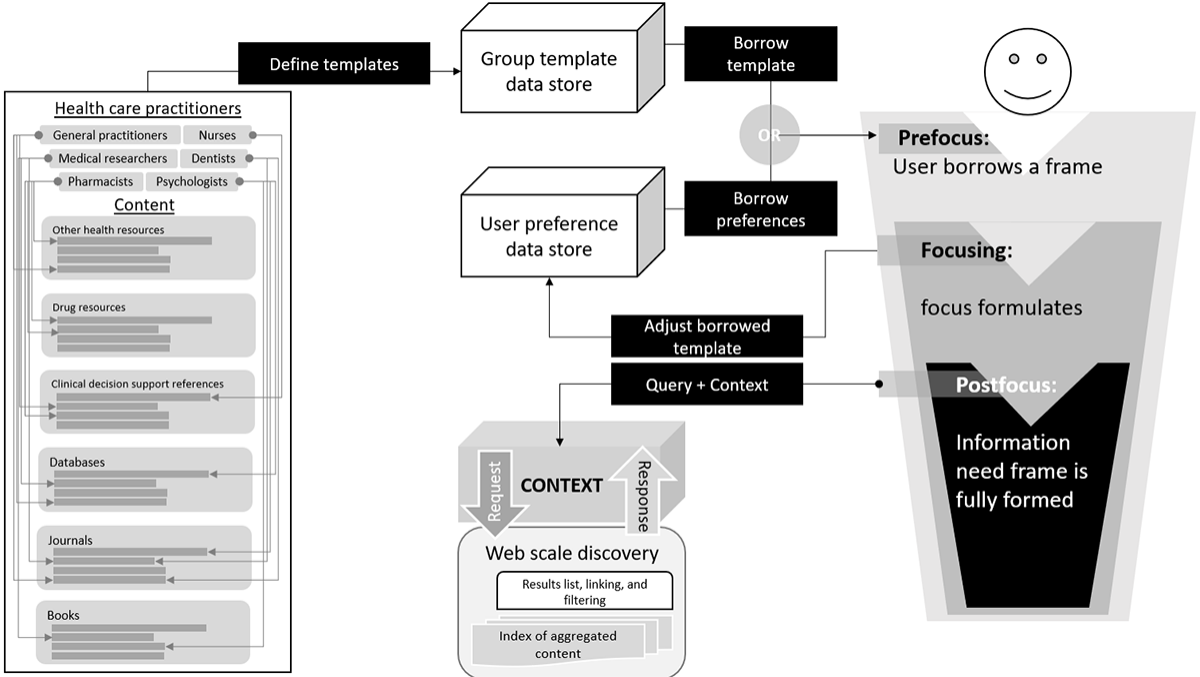
Artefact model to incorporate context when retrieving information from Web-scale discovery services.

Up to this point, this research study has described the need for EBP and how WSD services solved the problem of accessing research evidence as illustrated in Figures 2 and 3, but there are problems with the current services. Figure 4 proposed an improvement by applying a contextual layer to Cole’s model of a user-centered system instead of a computer science–focused system as illustrated in Figure 5. Figure 9 draws on gradual development of this theory to propose a contextual model to integrate with existing WSD services.

Starting from the rightmost end of the illustration, a user first borrows a frame for information retrieval for prefocus. For WSD services without the contextual model, the frame is an institutional level context that was configured as part of the WSD implementation. For WSD services that implement the contextual model, the frame can be further personalized to the user by allowing them to borrow a peer’s frame. Focusing deeper, a user’s focus formulates as part of the user’s own information needs until an information need is fully formed as postfocus to capture the context and submit it with the query to retrieve results that are applicable to the user’s context.

Developing the context, however, is a prefocus step where the model allows institutions to iteratively develop clinical group templates to borrow in the group template store by analyzing resource needs if a WSD was not available. These initial templates satisfy the adopt group context requirement of the model to help create a scope for information retrieval.

Individual users then have the option to personalize the template and define a more specific user context, which is stored in the user preference data store. This introduces persistence for context, so users do not have to define their context every time they initiate a new session on the WSD. The context is applied as soon as the system recognizes the user.

Data collection and analysis of the model was done through observation and refinements based on iterative prototyping [28]. Instantiations of the model were developed on the EDS [33] using its application programming interface (API) to iteratively prototype the context layer. A combination of Web technologies such as HTML, CSS, and JavaScript was used for the mobile first front-end. PHP was used for server-side, whereas Google Firebase [34] was used for authentication and storage.

On the basis of observations and comparison of results with and without the context layer, the model and subsequent instantiations were refined several times until it was observed that the model had an impact on information retrieved from the WSD to satisfy R1 to R5.

## Results

### Evaluation Overview

Once observations showed sufficient evidence that the prototype as a context layer satisfied R1 to R5 for information retrieved from a WSD compared with its noncontextual state, the instantiation was evaluated in the real world with select health care practitioners using convergent interviewing [30] for a summative evaluation followed by a confirmatory focus group consisting of health researchers.

### Demonstration

As per DSRM, demonstration was used to solve the problems, observe results, and develop an instantiation or prototype of the context model [2].

**Figure 10:**
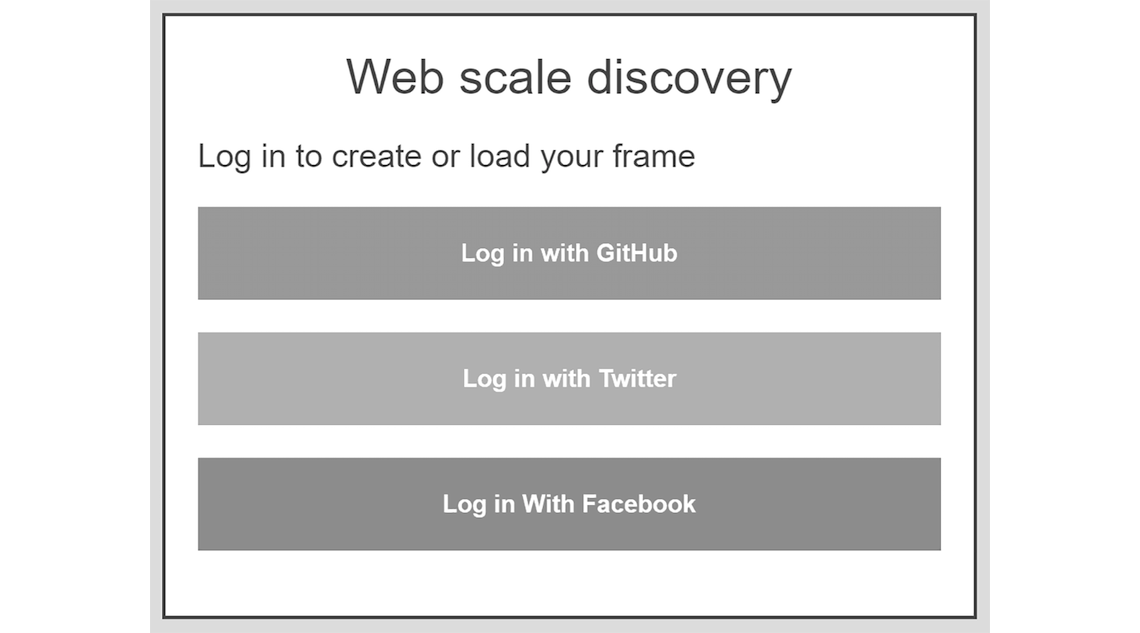
Login screen to recognize user and load existing or create new context.

The first step is to recognize the user. The developed prototype allows users to sign in using providers such as GitHub, Twitter, or Facebook shown in Figure 10. Once the user authorizes the instantiation to recognize the user, it checks to see if the user has already borrowed a frame or if the default institutional frame should be applied.

**Figure 11:**
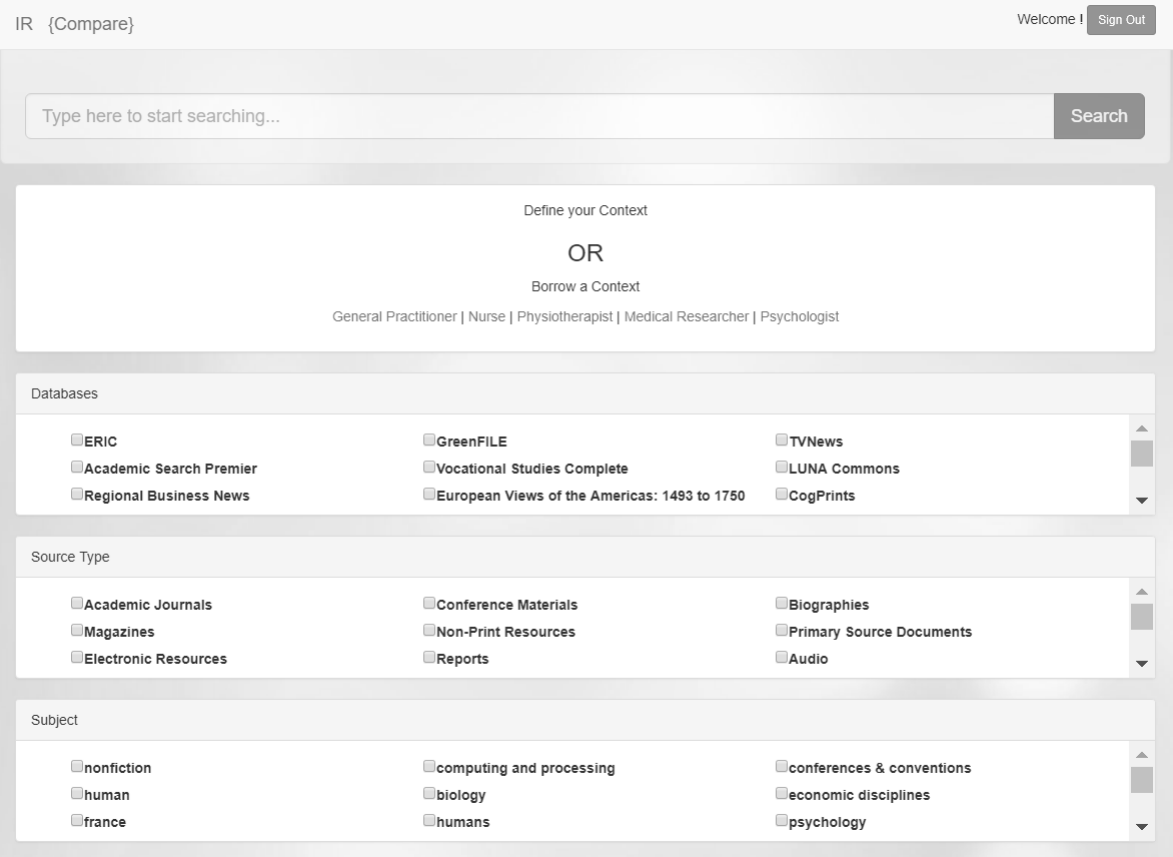
Screenshot of prototype showing options to define context or borrow an existing context.

The user is presented with all the filters that are available through the WSD API, and the user context is applied automatically if the frame was borrowed before. If the user logs into the instantiation for the first time, none of the filters are preselected, and the default frame is the institutional frame. User can choose to define their context or borrow a context from predefined frames. The prototype shown in Figure 11 includes frames for a general practitioner, nurse, physiotherapist, medical researcher, and psychologist. The model allows institutions to define and present any number of frames to borrow.

The settings enabled through the EBSCO WSD to define the context are databases, source type, subject, publication, and publisher. These settings will be different for WSD from other vendors.

It is not necessary for a user to define a full context right away, and this can be a gradual process as the prototype will store the selections for future reference allowing for gradual refinement of the context.

**Figure 12:**
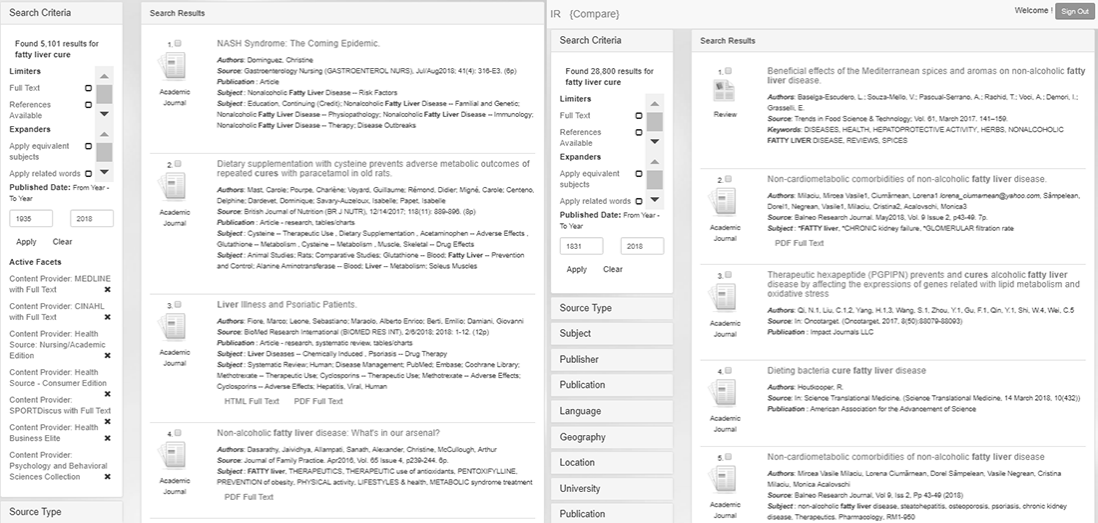
Screenshot of prototype comparing results with context (left) and without context (right).

The screenshot in Figure 12 shows a comparison between information retrieved from the prototype with the context layer (left) versus results without the context layer (right). This example borrows the general practitioner frame to submit the query “fatty liver cure.” The example with the context layer (left) retrieved 5101 results, and the context is visible under the active facets section. The same query without the context layer (right) returned 28,000 results using the institutional frame. The type of information retrieved is presented on screen for qualitative evaluation.

### Results of Artefact Evaluation

“The technical risk and efficacy evaluation strategy emphasizes artificial formative evaluations iteratively early in the process but progressively moving toward summative artificial evaluations” [29].

A key risk in the evaluation was to determine if a contextual layer could technically be implemented to improve WSD services, so the initial version of the model was developed using the theory of iterative prototyping to allow observations and refinements. Once data collection and observations showed that WSD information retrieval using the context layer satisfied R1 to R5 as part of artefact formative evaluations, a naturalistic summative evaluation was conducted using qualitative and quantitative research methods with health care professionals and health researchers, respectively.

#### Convergent Interviewing

Convergent interviewing [30] allows researchers to identify and select participants to interview them for key issues and finalize results from qualitative analysis. This technique targeted health care practitioners with a significant length of experience in the health care sector as medical practices has progressed significantly over the years making EBP invaluable. Their practical experience with research evidence to provide patient care using current relevant practices qualifies for qualitative naturalistic summative evaluation.

During the one-on-one interview, the 5 health care practitioners were informed about the nature of this study complying with ethical requirements. Once consent was obtained, a live demonstration of the instantiation was provided to the health care practitioners followed by questions to evaluate the prototype for its validity and utility. They were also able to compare the results against a WSD implementation without the context layer. The results are shown in Table 3, which maps interview questions asked against the requirements identified for this study.

On all 5 counts R1 to R5, the health care practitioners saw benefits and confirmed that the context model improved the information retrieval process using WSD services compared with not using a contextual layer. One of the doctors expressed keenness to have this implemented across their medical practice, while a professor who is also a registered psychologist highlighted the value this would have for his students.

**Table 3:** Results of prototype evaluation through convergent interviewing.

Requirements, Evaluation criteria, Evaluated by			Satisfied (Yes/No)	Comments (if any)
**Evaluating validity**					
	**R1^a^: Does the instance allow you as a clinician to select and configure your own settings for a personalized profile?**				
		Doctor 1	Yes	“The system gives features for defining my context with preferences.”
		Doctor 2	Yes	“Profiling seems ok to me but can we add any new details?”
		Doctor 3	Yes	“Professional preferences can be added that are supportive in finding quick resources.”
		Psychologist 1	Yes	“There are many settings for detailed selection.”
		Psychologist 2	Yes	“Several options not related to my area.”
	**R2^b^: Are you as a clinician able to borrow a predefined context for a clinical group you belong to or adopt another clinical group to see difference in context?**				
		Doctor 1	Yes	“Yes, it is a good way of seeing a comparison.”
		Doctor 2	Yes	“The difference can lead to a good outcome.”
		Doctor 3	Yes	“It is a learning by seeing the difference, although we don’t have time to check it during surgery time.”
		Psychologist 1	Yes	“Seeing different groups shows wide application across healthcare.”
		Psychologist 2	Yes	“I could select psychologist and other groups.”
	**R3^c^: Does the instance prioritize the resources you as a clinician would prefer to search using a Web-scale discovery?**				
		Doctor 1	Yes	“It is a good system to see quick and concise searching result.”
		Doctor 2	Yes	“Searching process seems simple enough.”
		Doctor 3	Yes	“It can bring me a to-the-point answer.”
		Psychologist 1	Yes	“Psychologist group pre-selected all the resources I would select generally.”
		Psychologist 2	Yes	No comment
	**R4^d^: Does the instance apply your defined context to reduce noise or irrelevant results to your particular case?**				
		Doctor 1	Yes	“I can see only very relevant resources.”
		Doctor 2	Yes	“Outcome of searching makes sense to particular aspect.”
		Doctor 3	Yes	“The application removes unnecessary resources.”
		Psychologist 1	Yes	“Removing irrelevant databases shows improvement.”
		Psychologist 2	Yes	“Better compared to results without selecting a group.”
		
**Evaluating utility**					
	**R5^e^: Does the instance improve the medical relevance of the Web-scale discovery compared with using it without clinical context?**				
		Doctor 1	Yes	“It offers better with context.”
		Doctor 2	Yes	“I’m happy to use this application as it is very work related.”
		Doctor 3	Yes	“The system supports with our work practices.”
		Psychologist 1	Yes	“Since only medical sources are selected.”
		Psychologist 2	Yes	“Saves a lot of time.”
	**R5^e^: Dvidence-based practice or EBP consists of patient values, clinical expertise, and research evidence. Does this instance add value to the research evidence dimension of a clinician doing EPB?**				
		Doctor 1	Yes	No comment
		Doctor 2	Yes	“I found it is helpful for our practice in the future please let us know we are very happy to provide more feedback.”
		Doctor 3	Yes	“It would be great if we could get the complete product for all of our GPs.”
		Psychologist 1	Yes	“Would benefit psychology students and new practitioners.”
		Psychologist 2	Yes	No comment

^a^R1: Define user context.

^b^R2: Adopt group context.

^c^R3: Prioritize resources.

^d^R4: Reduce noise.

^e^R5: Medical relevance.

#### Confirmatory Focus Group

Focus groups are used as an evaluation method in DSR and are an appropriate approach [35], which can either be exploratory in nature more aligned to formative evaluations or confirmatory used for summative evaluations [31]. In this case, a confirmatory focus group was formed to quantitatively analyze the discussion content to determine significant focus on requirements to justify the group’s conclusion.Content analysis is a research technique that explores data obtained directly from sources such as human interactions and written documents for research quantitatively or qualitatively or both [36].

Forming a confirmatory focus group requires selection relevant to the area of research [32], so 5 doctoral level health researchers with hands-on exposure to using a discovery service were invited to take part in the discussion. An initial introduction was provided to the group along with the 5 requirements R1 to R5, as shown in Table 2, that assessed other discover services followed by a demonstration of the model’s instantiation to discuss if it met all requirements.

Although there were comments about improving the detailed functionality of the prototype, such as the way the options screen could be more user-friendly and the limiting nature of the context to multidisciplinary content, the group consensus was that the instantiation satisfied all requirements for the artefact to have validity and utility.

Content analysis in the context of focus groups comprises forming a hypothesis to test through analysis. Semantical content analysis “which seeks to classify signs accordingly to their meaning,” in particular the subclassification assertion analysis “which provides the frequency with which certain objects are characterized in a particular way” [32], was used to test if the hypothesis that all requirements R1 to R5 were discussed significantly for the focus group to justify the conclusion that the artefact instantiation satisfied all the requirements.

For this analysis, the requirements R1 to R5 were identified as objects positioned as columns and codes or words with frequency positioned as rows in the resulting matrix.

To rigorously analyze the discussion and its correlation to the conclusion, the session was recorded and later transcribed by converting the audio recording to a video file and uploading to YouTube to auto-generate the transcript. The text was copied into a raw HTML file to open in a browser removing new lines and saved as a continuous stream in a text file.

The text file was imported into Microsoft Excel as a space separated data file and transposed so each word appeared in a row. After this, a visual basic script was developed to analyze each word and determine its parts of speech (eg, verb and noun) and populate the cell adjacent to the word. This Excel file with the word, part of speech, and word length as columns was then imported into Tableau Software’s Desktop tool for further analysis.

**Figure 13:**
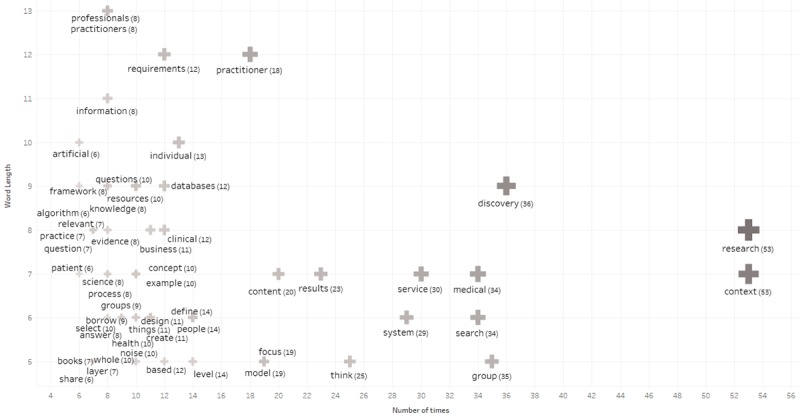
Result of content analysis descriptors from confirmatory focus group.

Inductive coding [32] was done using the imported data by creating a scatter plot using the number of times a word was mentioned in the discussion as x-axis and word length on y-axis for further interpretation. Select words belonging to parts of speech such as adverbs, conjunctions, interjections, pronouns, and other were removed leaving only adjectives, nouns, and verbs. Words with length less than 5 characters were ignored including words that were mentioned less than 6 times. The scatterplot was further analyzed word for word to selectively remove obscure words such as apply, being, and possible that did not apply to the research context, leaving the scatter plot with descriptors or codes to present outcomes shown in Figure 13.

Words with their frequency count (descriptors or codes) were analyzed for signs of association with 1 of the requirements R1 to R5 (objects) to form a matrix Table 4 consisting of top 5 descriptors per object to demonstrate that significant discussion for all requirements took place to confirm utility and validity of the artefact to declare the hypothesis as true.

**Table 4:** Results of assertion analysis.

R1^a^	R2^b^	R3^c^	R4^d^	R5^e^
context (53)	group (35)	focus (19)	noise (10)	medical (34)
individual (13)	level (14)	content (20)	relevant (7)	health (10)
person (7)	select (10)	resources (10)	results (23)	clinical (12)
create (11)	borrow (9)	knowledge (8)	discovery (36)	practitioner (18)
Define (14)	share (6)	information (8)	research (53)	databases (12)

^a^R1: Define user context.

^b^R2: Adopt group context.

^c^R3: Prioritize resources.

^d^R4: Reduce noise.

^e^R5: Medical relevance.

## Discussion

### Meeting Requirements

Using a combination of artificial formative evaluations based on iterative prototyping followed by naturalistic summative evaluation using convergent interviewing and confirmatory focus group, the context model satisfied all requirements R1 to R5 of this research.

This research reviewed the state of EBP by health care practitioners and identified that the research evidence dimension was a significant barrier because of the vast myriad of resources. WSD services facilitated research evidence by indexing most licensed content while offering practitioners a single point of entry into research; this solved the problem to a certain degree.

The problems with this approach as identified in the literature was the lack of relevancy to the medical field, creation of noise, unprioritized resources, and missing user context. These problems were synonymous with information retrieval that were developed with a computer science approach focusing on systems and algorithms instead of user need.

On the basis of the theory of information need by Cole, this research designed and evaluated a contextual layer or artefact model to apply to WSD services to improve them. Using DSRM principles, this research used iterative prototyping to observe, design, and refine the model and its prototype against the EBSCO WSD using its APIs.

Once the prototype demonstrated that it satisfied requirements R1 to R5 during a series of artificial formative evaluations, 5 health care practitioners were selected for convergent interviewing for a qualitative naturalistic summative evaluation.

### Principal Findings

Outcomes from this research provided very positive views about the artefact as all health care practitioners who participated in the field experiments found the proposed approach useful, compatible to their own practices, and added value to WSD services to improve EBP.

A quantitative naturalistic summative evaluation was also performed by forming a confirmatory focus group consisting of 5 health researchers who discussed and concluded that the artefact instantiation met all the requirements. The discussion was recorded and transcribed for content analysis to identify descriptors or codes and associate them to objects or requirements to form Table 4 for a positive hypothesis that there was evidence of significant discussion across the requirements to justify the conclusion.

Evaluation is one of the crucial elements of DSR as it provides feedback to further improve the artefact and bring it to a state where its utility, quality, and efficacy are validated [26].

Prior research [29] shared strategies for DSR evaluation, namely, quick and simple, human risk and efficacy, technical risk and efficacy, and the purely technical artefact strategy. The choice of evaluation depends on the functional purpose of the artefact, which in this research is formative as it aims to improve the outcomes of the process under evaluation. It is also summative as the outcomes will be judged to what extent they match expectations. The other factor for choice of strategy is the evaluation paradigm: if it is artificial or naturalistic.

In this dimension, the paradigm is both artificial and naturalistic as the context artefact is evaluated using iterative prototyping to determine if the context is feasible with a WSD as part of its development. This guided the development in an incremental and iterative fashion to address the research problem. Here, the prototype design and development effort is used to validate or invalidate the theory [28].

### Conclusions

The evaluation concluded that the context layer or artefact model had validity and utility as an artefact to solve problems with WSD for EBP. Rigorous research in DSR [29] showed that formative evaluation is necessary to identify weaknesses and areas of improvement during artefact development, and summative evaluation is important to conduct the DSR study by outlining overall utilities or efficiencies and potential benefits of the proposed artefact. Considering artificial evaluation via conducting iterative prototyping and naturalistic summative evaluation, the technical risk and efficacy evaluation strategy was an appropriate fit for this research.

Further research into the contextual approach is recommended to evaluate the artefact using the human risk and effectiveness strategy.

## Abbreviations

API: application programming interface
DSR: design science research
DSRM: design science research methodology
EBP: evidence-based practice
EDS: EBSCO Discovery Service
e-resources: electronic resources
OCLC: Online Computer Library Center
WSD: Web-scale discovery
